# Complete chloroplast genome of *Callicarpa formosana* Rolfe, a famous ornamental plant and traditional medicinal herb

**DOI:** 10.1080/23802359.2020.1820399

**Published:** 2020-09-21

**Authors:** Yongxi Du, Yanfeng Liu, Bo Liu, Tielin Wang

**Affiliations:** a National Resource Center for Chinese Meteria Medica, China Academy of Chinese Medical Sciences, Beijing, P. R. China; b State Key Laboratory for Biology of Plant Diseases and Insect Pests, Institute of Plant Protection, Chinese Academy of Agricultural Sciences, Beijing, P. R. China

**Keywords:** *Callicarpa formosana*, chloroplast genome, phylogenetic relationship

## Abstract

*Callicarpa formosana* is a species of beauty-berry with large medicinal value belonging to the family Verbenaceae. In this study, the complete chloroplast genome of *C. formosana* was sequenced using Illumina Hiseq X Ten platform. The chloroplast genome was 1,54,210 bp in length, containing two short inverted repeat (IRa and IRb) regions of 25,701 bp, which was separated by a large single copy (LSC) region of 84,938 bp and a small single copy (SSC) region of 17,870 bp. The GC content of the whole chloroplast genome was 38.1%. The chloroplast DNA of *C. formosana* comprised 113 genes, including 79 protein-coding genes, 4 ribosomal RNA genes, and 30 transfer RNA genes. Phylogenetic analysis indicated that the genus *Callicarpa* L. was located in the basal position within the family Verbenaceae. The chloroplast genome **(**cpDNA) of *C. formosana* was closely related to *Callicarpa nudiflora*.

*Callicarpa* L. is a genus of shrubs and small trees in the family Verbenaceae. In recent years, research on the medicinal value of *Callicarpa* plants has increased. Four species of *Callicarpa* were included in the Pharmacopeia of the People’s Republic of China. *Callicarpa formosana* Rolfe is a species of beauty-berry. It is native to China (type specimens were collected from Taiwan), Japan, and Philippines (Wu et al. 1993–2013; Nakashima et al. 2016). The species is cultivated as an ornamental plant that is adaptable to various conditions of cultivation and climates. The globous drupes of resembling tiny clusters of berries are of lavender color. All parts of the plant are utilized in the traditional Chinese medicine for various pathologies (National Pharmacopeia Committee 2020). Chloroplast genomes are important sources for phylogenetic analyses, genetic diversity evaluation, and plant molecular identification (Dong et al. 2018; Sun et al. 2020). In this study, we determined the complete chloroplast genome (cpDNA) sequence of *C. formosana* based on the next-generation sequencing method. The annotated cpDNA has been deposited into GenBank with the accession number MT830861.

Fresh samples of *C. formosana* were collected from Suining county, Hunan province, China (26°36′16′′N, 110°8′18′′E). Voucher specimen was deposited at the herbarium of Institute of Chinese Materia Medica (CMMI), China Academy of Chinese Medical Sciences with the specimen voucher number is 430527LY0206. Total genomic DNA from fresh leaves of a single individual was isolated using the method of Li et al. (2013). And the sequencing library was constructed and quantified following the methods introduced by Dong et al. (2017). The whole genome sequencing was conducted with 150 bp paired-end reads on the Illumina HiSeq X Ten platform. Next-generation sequencing QC toolkit was used for quality control and to filter the low quality reads. Contigs were assembled from the high quality paired-end reads by using the SPAdes version 3.6.1 program (Kmer = 95) (Bankevich et al. 2012). The chloroplast genome contigs selected by the Blast program (Altschul et al. 1990), taken *Callicarpa nudiflora* (GenBank: MK783316) as the reference. The selected contigs were assembled using Sequencher version 4.10 (Gene Codes Corporation, Ann Arbor, MI USA, http://www.genecodes.com). Gene annotation of *C. formosana* was performed using DOGMA annotation (Wyman et al. 2004) and manually corrected for codons and gene boundaries using BLAST searches.

The circular cpDNA of *C. formosana* was 1,54,210 bp in length, containing two short inverted repeat (IRa and IRb) regions of 25,701 bp, which was separated by a large single copy (LSC) region of 84,938 bp and a small single copy (SSC) region of 17,870 bp. The GC content of the whole chloroplast genome was 38.1%. The cpDNA of *C. formosana* comprised 113 distinct genes, including 79 protein-coding genes, 4 ribosomal RNA genes, and 30 transfer RNA genes. In these genes, 19 were duplicated in the IR regions and 19 genes contained one or two introns.17 harbored a single intron, and two (*ycf3*、*clpP*) contained double introns.

Eighteen chloroplast genome sequences were used for phylogenetic analysis to confirm the location of *C. formosana*, including four outgroup samples and 14 samples of Verbenaceae from the GenBank. We used 79 protein-coding genes to conduct a maximum likelihood (ML) analysis using IQ-tree under the GTR + G model with 1000 bootstrap replicates (Nguyen et al. 2015; Zhang et al. 2020). The phylogenetic analysis revealed that samples of Verbenaceae were strongly supported as monophyletic, the genus *Callicarpa* was located in the basal position additionally (Figure 1). The cpDNA of *C. formosana* is closely related to *C. nudiflora*. The complete chloroplast genome reported in this study will be a valuable resource for future studies on genetic diversity, taxonomy, and phylogeny of family Verbenaceae.

**Figure 1.**
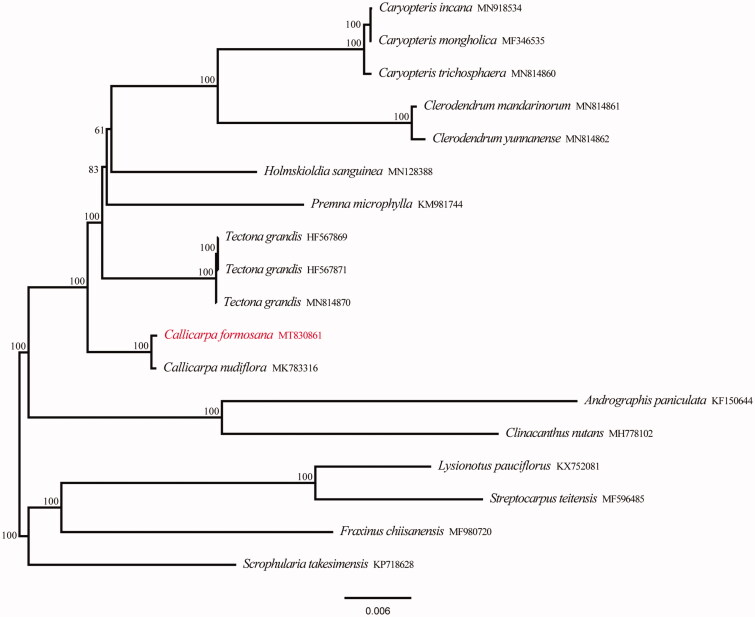
Phylogenetic tree reconstruction of 18 taxa using maximum likelihood (ML) methods based on protein-coding genes in the chloroplast genome sequences. ML bootstrap support value presented at each node.

## Data Availability

The data that support the findings of this study are openly available in GenBank of NCBI https://www.ncbi.nlm.nih.gov/, reference number MT830861, raw data submissionID: SUB7932473, BioProject ID: PRJNA657909.
